# Inflammatory loops in the epithelial–immune microenvironment of the skin and skin appendages in chronic inflammatory diseases

**DOI:** 10.3389/fimmu.2023.1274270

**Published:** 2023-09-28

**Authors:** Teruki Dainichi, Masashi Iwata

**Affiliations:** Department of Dermatology, Kagawa University Faculty of Medicine, Kagawa, Japan

**Keywords:** EIME, atopic dermatitis, psoriasis, systemic lupus erythematosus, alopecia areata, and acne

## Abstract

The epithelial–immune microenvironment (EIME) of epithelial tissues has five common elements: (1) microbial flora, (2) barrier, (3) epithelial cells, (4) immune cells, and (5) peripheral nerve endings. EIME provides both constant defense and situation-specific protective responses through three-layered mechanisms comprising barriers, innate immunity, and acquired immunity. The skin is one of the largest organs in the host defense system. The interactions between the five EIME elements of the skin protect against external dangers from the environment. This dysregulation can result in the generation of inflammatory loops in chronic inflammatory skin diseases. Here, we propose an understanding of EIME in chronic skin diseases, such as atopic dermatitis, psoriasis, systemic lupus erythematosus, alopecia areata, and acne vulgaris. We discuss the current treatment strategies targeting their inflammatory loops and propose possible therapeutic targets in the future.

## 1 Introduction

**Figure 1:**
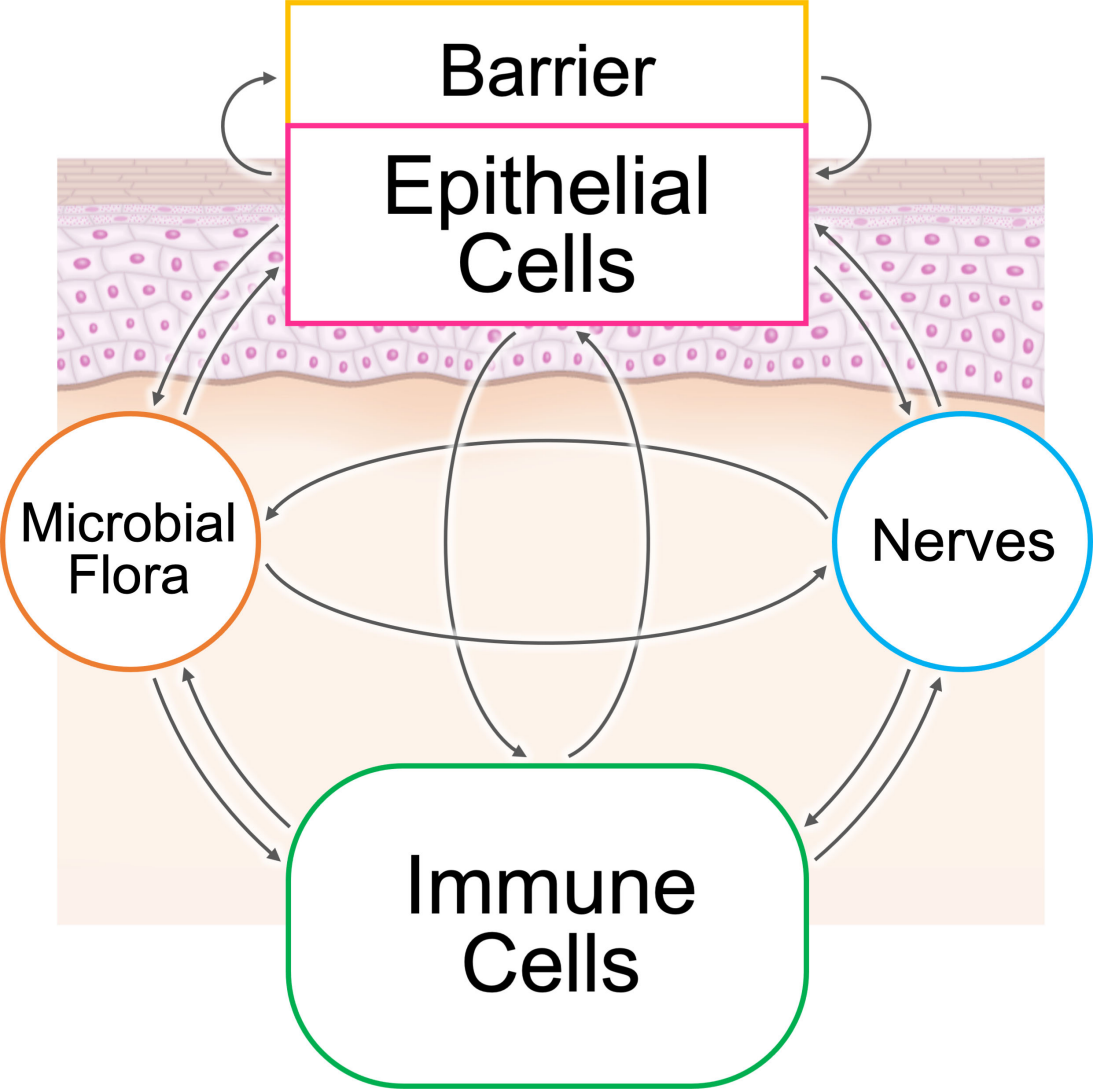
The epithelial–immune microenvironment (EIME) of the skin and skin appendages. There are five common elements in the microenvironment of epithelial tissues: microbial flora, barrier, epithelial cells, immune cells, and peripheral nerve endings. The interaction between these five elements provides protection against dangers from the environment.

The epithelial–immune microenvironment (EIME) provides both constant defense and situation-specific protective responses in several organs, such as the skin, gut, and lungs, which are located at the interface between the environment and an organism (1). The host defense system can be classified into three layers: (constant and nonspecific) barriers, innate immunity, and acquired immunity (2). There are five common elements in the microenvironments of these organs: microbial flora, barriers, epithelial cells, immune cells, and peripheral nerve endings (**Figure 1**). The interaction between these five elements provides protection against dangers from the environment. This dysregulation can result in the generation of inflammatory loops in chronic inflammatory diseases (1).

The skin is one of the largest organs in the host defense system (3). Here, we propose an understanding of the EIME in five chronic skin diseases: atopic dermatitis (AD), psoriasis, systemic lupus erythematosus (SLE), alopecia areata (AA), and acne vulgaris. We discuss current treatments targeting inflammatory loops and propose possible therapeutic strategies for the future.

## 2 Loops in atopic dermatitis

Atopic dermatitis (AD) is a common chronic inflammatory skin disease characterized by chronic pruritic eczematous skin lesions (1). AD is an atopic disorder characterized by elevated serum concentrations of immunoglobulin E (IgE) (4). AD has two age peaks (infancy and the third decade of life) in its prevalence and is spontaneously ameliorated (1). The onset of AD is often followed by serial occurrence of allergic diseases that represent the atopic march (5). AD lesions affect predilection sites including the cubital and popliteal fossae that are predominantly colonized by *Staphylococcus aureus* (1). Topical therapies with moisturizers and corticosteroids are first-line treatment (6). The blockade of interleukin (IL)-4 or IL-13 is highly effective, indicating that T_H_2-type inflammation is essential for its pathogenesis (1).

**Figure 2:**
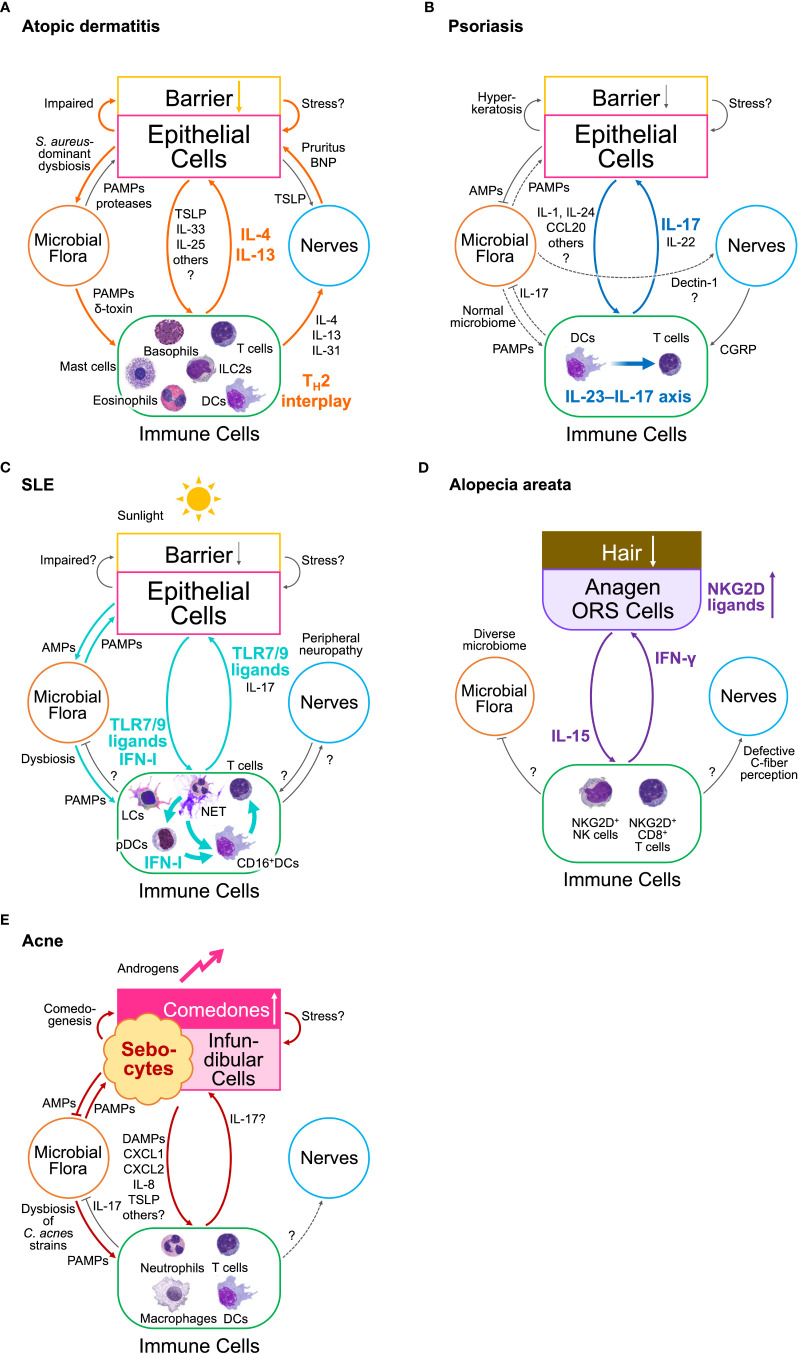
Inflammatory loops in the epithelial–immune microenvironment (EIME) of chronic inflammatory diseases **(A)** Loops in atopic dermatitis. Two inflammatory loops drive type 2 EIME in skin lesions in AD. One is the loop between epithelial and immune cells, which constructs T_H_2 interplay. The other is a loop involving *S. aureus*-dominant dysbiosis and abnormal sensory nerve endings that cause pruritus. **(B)** A loop in psoriasis. A single inflammatory loop between epithelial and immune cells in the interleukin (IL)-23–IL-17 axis drives type 17 EIME in lesional skin in psoriasis. The skin microbiome remains unchanged, suggesting less involvement of microbial flora, whereas *C albicans* colonization elicits a type 17 response by directly stimulating the sensory nerve endings. **(C)** Loops in systemic erythematosus (SLE). An inflammatory loop between epithelial cells, microbial flora, and immune cells is drawn in the EIME of lesional skin in SLE. Another loop may be organized without microbial flora. Keratinocyte damage caused by sunlight or microbial flora can trigger these loops. The plasmacytoid dendritic cells (pDCs) promote these loops only during the initiation phase by releasing type I interferons (IFN-I). The constitutive activation of Toll-like receptor (TLR)7/9 drives type-I IFN loops. Neutrophil extracellular traps (NET) activate pDCs at an early stage and promote disease propagation. **(D)** A loop in alopecia areata (AA). A single inflammatory loop of interferon (IFN)-γ and IL-15 is driven by the EIME of hair follicles (HFs) in the anagen phase. The outer root sheath (ORS) cells of HFs express abnormal or ectopic major histocompatibility complex (MHC) molecules and NKG2D ligands. IFN-γ produced from NKG2D^+^ T cells and NK cells induce hair loss and promote the expression of these molecules, and IL-15, from the HF ORS cells. IL-15 activates IFN-γ-producing cells. **(E)** Loops in acne vulgaris. The inflammatory loops in acne vulgaris involve sebocytes, infundibular cells, *Cutibacterium acnes*, and immune cells. An increase in androgen levels triggers these loops. Comedogenesis is a bottleneck in acne pathophysiology. AMPs, antimicrobial peptides; BNP, brain-derived natriuretic peptide; CCL, C-C motif ligand; CGRP, calcitonin gene-related peptide; CXCL, C-X-C motif ligand; DCs, dendritic cells; IFN, interferon; IL, interleukin; ILC, innate lymphoid cell; LCs, Langerhans cells; LTC_4_, leukotriene C4; NET, Neutrophil extracellular trap; NK, natural killer; ORS, outer root sheath; PAMPs, pathogen-associated molecular patterns; pDCs, plasmacytoid dendritic cells; T_H_2, T helper type 2; TLR, Toll-like receptor.

A relationship chart of the elements in type 2 EIME of AD depicts double loops (**Figure 2A**) (1). This redundancy results in the partial efficacy of IL-4/13 blockade therapies in AD, in contrast to the almost perfect efficacy of the IL-17-blockades in psoriasis. The first is a positive feedback loop between keratinocytes and immune cells. Keratinocytes produce epithelial type 2 mediators including thymic stromal lymphopoietin (TSLP), IL-33, granulocyte–macrophage colony-stimulating factor (GM-CSF), and IL-25. In contrast, type 2 cytokines, such as IL-4 and IL-13, produced by immune cells, activate keratinocytes via IL-4/13 receptors. The other is a positive feedback involving dysbiosis of the microbial flora and peripheral nerve sensing of pruritus. Impaired barrier formation in the skin results in *S. aureus*-predominant dysbiosis in AD. *S. aureus* activates type 2 immune responses. In contrast, IL-4 and IL-13 directly dampen barrier formation via IL-4/13 receptors in keratinocytes, and several type 2 cytokines indirectly damage the skin barrier by activating sensory nerve endings via receptors for IL-4, IL-13, IL-31, IL-33, and TSLP that cause pruritus and subsequent scratching behavior (7). Additional activation of G-protein-coupled receptors (GPCRs) and ion channels in sensory nerve endings may be involved in the itch–scratch cycle in AD (7).

IL-31 from immune cells enhances the release of brain-derived natriuretic peptide (BNP) from dorsal root ganglionic neurons (DRGs). BNP induces the activation of glycogen synthase kinase 3 (GSK3) and production of matrix metalloproteinase (MMP)9 in cultured human keratinocytes (8). These results suggest that the activation of sensory nerves directly affects keratinocyte activation and may impair the skin barrier, regardless of the induction of scratching behavior in the EIME of AD.

Basophils are involved in both chronic itch and itch flares in AD (9). In chronic AD skin lesions, keratinocytes produce TSLP that primes basophils to release IL-4, and activation of IL-4 receptors in sensory neurons drives chronic itch. In contrast, during allergen-stimulated AD itch flares, the epithelial barrier disruption allows increased allergen infiltration. IgE-R^+^ basophils recruited to the skin release leukotriene C4 (LTC_4_) and drive itch sensations via LTC_4_ receptors in sensory nerve endings (10).

Keratinocytes play a pivotal role in driving the inflammatory loop of type 2 inflammation in AD (1). Single-cell RNA sequencing of skin lesions from patients with AD who underwent long-term treatment with the IL-4Rα blocker dupilumab demonstrated that transcriptomic dysregulation in keratinocytes was completely normalized, whereas the AD signature in dendritic cells (DCs) and T lymphocytes persisted for up to a year after clinical remission (11). These results suggest that keratinocytes are the major target of dupilumab in AD, and that IL-4/13 signaling in keratinocytes is essential for the inflammatory loop of type 2 EIME in AD, regardless of the persistent activation of DCs and T lymphocytes.

## 3 Loops in psoriasis

Psoriasis is a common chronic inflammatory disease characterized by both cutaneous and systemic manifestations (1, 12). It is clinically characterized by red scaly papules and plaques, and can be associated with psoriatic arthritis. Its prevalence is estimated to be 1–3% worldwide. Psoriasis typically develops in genetically predisposed middle-aged individuals and is commonly associated with metabolic syndrome. Genetic predisposition is related to keratinocyte pro-inflammatory signaling and type 17 responses. The efficacy of selective biologics targeting tumor necrosis factor (TNF), IL-23, and IL-17 has demonstrated their pivotal roles in the pathogenesis of psoriasis (1).

Psoriasis simulates the protective machinery of the body opposing dermatophytes. The skin removes them together with the stratum corneum by accelerating its turnover and neutrophil attacks, mediated by the T_H_17 response, which is called ‘psoriasiform dermatitis,’ characterized by epithelial hyperplasia and neutrophil infiltration (1).

p38 mitogen-activated protein kinase (MAPK)-dominant activation of the TNF receptor–associated factor 6 (TRAF6) pathway in keratinocytes may be involved in triggering psoriasis (13). Many psoriasis-susceptibility genes, such as *IL36RN* and *CARD14*, are related to skin-specific p38 activation. In addition, psoriasis develops during middle age, and the p38 pathway activation in the skin of aged individuals is more inducible than that of young subjects (14). Furthermore, skin scrubbing elicits psoriatic lesions (Koebner’s phenomenon), and physiologically scrubbed stresses immediately induce p38 activation in keratinocytes (15). Moreover, keratinocyte TRAF6 signaling is necessary for releasing proinflammatory cytokines and chemokines, such as IL-1, IL-6, C-X-C motif ligand (CXCL)1, and C-C motif ligand (CCL)20, and for the activation and propagation of the IL-23–IL-17 axis in psoriatic inflammation (16), while the cutaneous p38 activation is sufficient to induce psoriatic inflammation (15).

In contrast to the type 2 EIME in AD, the type 17 EIME in psoriasis depicts a single-loop circuit (**Figure 2B**) (1). This is consistent with the efficacy of biologics targeting IL-17, IL-23, and TNF in this loop in type 17 EIME in psoriasis. transient receptor potential vanilloid 1 (TRPV1)^+^ sensory nerves sense *Candida albicans* and drives type 17 protective cutaneous immunity (17). By contrast, microbiota-induced *S. aureus*-specific T_H_17 cells accelerate sensory neuronal regeneration (18). However, the fungal and bacterial skin microbiota in lesional skin of patients with psoriasis are similar to those in non-lesional or healthy skin (1). Therefore, despite the bidirectional interaction between skin microbiota and sensory nerves in an acute protective response (17, 18), these two elements do not appear to contribute to the formation of a closed circuit between other elements in the EIME during chronic inflammation. Collectively, the contribution of skin microbiota and sensory nerves to the inflammatory loop in type 17 EIME remains obscure in psoriasis.

## 4 Loops in systemic lupus erythematosus

SLE is a systemic syndrome that affects multiple organs including the skin, kidneys, brain, and vasculature, with a profound clinical heterogeneity (19–21). SLE has long been considered a systemic autoimmune disease. However, recent progress suggests that the initial trigger probably involves recognition of self or foreign molecules, especially nucleic acids, by innate sensors (22).

SLE can be spontaneously triggered by exposure to environmental stimuli such as ultraviolet light or infection (21). Dysregulation of apoptosis and nuclear debris clearance is a characteristic of SLE and contributes to multi-organ autoimmunity (23). Studies in mice and humans have shown definitive roles of neutrophils, plasmacytoid DCs (pDCs), Toll-like receptor (TLR) activation, and type I interferon (IFN) production in SLE, and increased IL-17 production may contribute to this process (20).

The skin of patients with SLE shows LC defects and reduced epidermal epidermal growth factor receptor (EGFR) phosphorylation, and topical EGFR ligands reduce photosensitivity (24). These results suggest that a defective Langerhans cell–keratinocyte axis protects against photosensitivity and triggers keratinocyte apoptosis and subsequent events in SLE.

SLE patients display an increased capacity to form neutrophil extracellular traps (NETosis). NETs harboring self- and foreign RNA and DNA antigens are poorly cleared and stimulate pDCs to produce type I IFN via TLR7 and TLR9 stimulation. It induces an innate immune response and the propagation of proinflammatory T_H_17 cells that are involved in disease expression and promote NETosis (20). The blockade of type I IFN receptor by treatment with anifrolumab is effective for reducing the disease activity in patients with SLE (25).

In patients with cutaneous lupus erythematosus (CLE), interfollicular keratinocytes exhibit a type I IFN–rich signature in pre-lesional skin (26). pDCs dominated the perifollicular region in non-lesional skin but not in lesional skin. In contrast, CD16^+^ DCs arise from non-classical monocytes, migrate into the non-lesional skin, and undergo IFN education for inflammation in the CLE.

TLR7 gain-of-function gene mutations and single nucleotide polymorphisms (SNPs) in the TLR trafficking chaperone UNC93B1 are found in patients with SLE (27, 28). Epicutaneous application of TLR7 agonists for four weeks led to a significant increase in *Ifna* expression in the spleen and the development of SLE-like systemic autoimmunity (29). These results indicate that dysregulation of EIME in the skin results in systemic autoimmunity.

Gut barrier defects associated with microbial dysbiosis have been observed in SLE patients and mouse models (30, 31). Additionally, the skin microbiota of patients with SLE is distinct from that of healthy individuals (32, 33). Notably, *S. aureus* skin colonization in epithelial cell–specific IκBζ-deficient (*Nfkbiz^ΔK5^*) mice promotes SLE-like autoimmune inflammation via caspase-mediated keratinocyte apoptosis and the subsequent activation of neutrophils and the IL-23–IL-17 axis (34).

Among patients with SLE, 7.6% experience peripheral nervous system events, including peripheral neuropathy (35). SLE may have an early effect on peripheral nerve function in patients without clinical or electrophysiological neuropathy (36). However, little is known about the interactions among peripheral nerves in the EIME of the skin. Peripheral blood mononuclear cells (PBMCs) from SLE patients are highly susceptible to apoptosis induced by calcitonin gene-related peptide (CGRP), a neuropeptide produced by the central and peripheral nerves (37). CGRP from the peripheral nerves drives dermal DCs to produce IL-23 in a type 17 response to cutaneous *C. albicans* infection (17). Notably, increased serum levels of procalcitonin, an alternative transcription product of CGRP, are diagnostic markers of bacterial infection in patients with SLE (38).

Thus, an inflammatory loop of type I IFN between keratinocytes and immune cells emerges in the EIME of the skin in patients with SLE (**Figure 2C**). Increased susceptibility to keratinocyte cell death induces the release of danger-associated molecular patterns (DAMPs), neutrophil recruitment, and NETosis, which trigger the activation of the TLR7 and TLR9 pathways in pDCs and their production of type I IFN. The type I IFN-rich signature of the skin primes CD16^+^ DCs and propagates a type 17 immune response involving keratinocyte activation and NETosis. However, the contribution of dysbiosis and peripheral neuropathy and the difference in the role of the type 17 immune response in psoriasis and SLE remain obscure.

## 5 Loops in alopecia areata

Alopecia areata (AA) is a common, acquired, non-scarring hair loss that affects 2% of the global population and is intractable in severe and relapsing cases (39).

AA is an autoimmune disease resulting from a disruption in hair follicle immune privilege, a structure or a system that protects vital organs, including the central nervous system, testes, placenta, eyes, and hair follicles (HFs), from the potential harm of immune recognition (40). Immune-privileged sites in HFs prevent natural killer (NK) cells from activating them. Specifically, suppressed expression of major histocompatibility complex (MHC) class I and NKG2D ligands in healthy HFs protected them from NK cell attack and subsequent hair loss. In contrast, HFs in patients with AA show abnormal expression of major histocompatibility complex (MHC) class I and II molecules and NKG2D ligands. Indeed, a genome-wide association study (GWAS) in 1,024 patients with AA and 3,278 controls identified *ULBP3*, which encodes an NKG2D ligand, as the responsible gene (41). Histologically, late anagen HFs in patients with AA show perifollicular infiltration of mononuclear cells, including CD4^+^ or CD8^+^ NKG2D^+^ T cells and CD56^+^ NKG2D^+^ NK cells (42).

In AA, an inflammatory loop of IFN-γ and IL-15 emerges between immune cells and HF epithelial cells in the EIME of the lesional scalp, and is thought to be the driving force of the disease state (**Figure 2D**) (43, 44). IFN-γ induces abnormal expression of MHC molecules and NKG2D ligands in the anagen hair bulb, leading to the collapse of the HF immune privilege. IFN-γ also acts on the HF epithelial cells to enhance the expression of IL-15. The expression levels of IL-15 and IL-15 receptor α in the outer root sheath of HF were higher in patients with AA and in animal models than in healthy controls (45). Furthermore, IL-15 signaling enhances CD8^+^ memory T cell survival, expansion, and maintenance of T and NK cells, and CD8^+^ T cell production of IFN-γ (46, 47). Consistently, serum levels of IFN-γ and IL-15 are higher in patients than in controls and correlate with disease activity (46, 47). In contrast, IL-15 prolongs anagen phase, stimulates proliferation, and suppresses apoptosis in the hair matrix of human scalp hair follicles (48). Of note, the IFN-γ pathway depends on Janus kinase (JAK)1/2 and the IL-15 pathway depends on JAK1/3, respectively (43, 44). Therefore, peroral JAK inhibitors selective for either JAK1/3 provide a clear example of the treatment development process via the blockade of the inflammatory loop in EIME (44, 49, 50).

The microbial flora of the lesional scalp may be less involved in AA pathogenesis because the scalp microbiome is more diverse in patients with AA than in healthy controls, but is not significantly different according to the severity of AA (51).

The AA scalp shows defective C-fiber sensory perception (52). However, the involvement of sensory nerves in the EIME of patients with AA remains largely unknown.

## 6 Loops in acne vulgaris

Acne vulgaris is a chronic inflammatory condition involving the pilosebaceous units of skin on the face, neck, chest, or back (53). Acne vulgaris affects approximately 85% of people aged 12–24 years, 18% of women, and 8% of men aged ≥ 25 years. Acne accounts for approximately 16% of the dermatological disease burden (54), and the global market size is estimated at USD 10.48 billion in 2022 (55). GWAS identified the possible link to genes related to androgen metabolism, inflammation processes, the tumor growth factor-β (TGF-β) pathway, and hair follicle development (56–58). A possible relationship between acne and diet, such as a high-glycemic-load diet or chocolate, has been suggested (59–61).

The development of acne involves the interplay of multiple factors, including (i) hormonal influences on sebum production and composition, (ii) follicular hyperkeratinization, and (iii) inflammation involving colonization with *Cutibacterium acnes* (62, 63). The specific relationship between these key factors remains to be defined, although an older study suggested that inflammation precedes hyperkeratinization (64).

Sebum production by the sebaceous glands is regulated by many factors and is primarily controlled by androgens that are produced both outside (gonads and adrenal glands) and inside the pilosebaceous unit (62, 63). Androgen levels are elevated during the neonatal period and puberty and have a significant impact on triggering the development of acne. Acne is associated with alterations in sebum composition. The sebum of patients with acne contains fewer essential free fatty acids (65) and increased levels of monounsaturated fatty acids (MUFAs) and lipoperoxides that influence keratinocyte proliferation and differentiation compared to healthy people (66–68). Notably, the topical application of fatty acids is sufficient to facilitate follicular hyperkeratosis in animal model (69).

Comedo, a hyperkeratotic plug in the infundibulum, is a diagnostic clue to acne vulgaris and can differentiate it from other acneiform eruptions. Microcomedo is the precursor of all acne lesions and a bottleneck in acne formation (62). However, the inciting event for microcomedo formation remains obscure whereas IL-1α may be involved (64).

*C. acnes* is a Gram-positive anaerobic/microaerophilic rod and is a commensal organism in the pilosebaceous unit. The amount of *C. acnes* is similar between patients with acne and healthy controls and was not correlated with disease severity; however, the strain populations differed between patients with acne and healthy individuals (70, 71). *C. acnes* and associated lipopolysaccharide (LPS) activate the TLR2/4 pathway and nucleotide-binding domain, leucine-rich–containing family, pyrin domain–containing-3 (NLRP3) inflammasome and induces the release of proinflammatory mediators, such as IL-8, TNF, IL-1α, IL-1β, and GM-CSF in human sebocytes and keratinocytes. In addition, *C. acnes* promote T_H_17 and T_H_1 response pathways, which are activated in acne lesions, by inducing the secretion of IL-17A and IFN-γ from CD4^+^ T cells (63). The type 17 immune response can affect keratinocyte proliferation and differentiation at the infundibulum and sebaceous duct in the pilosebaceous unit, and promote the infiltration of neutrophils via the release of their chemoattractants.

The facial skin of patients with acne is highly innervated and the sebaceous glands express receptors for several neuropeptides. Their activation in human sebocytes modulates cytokine production, cell proliferation, cell differentiation, lipogenesis, and androgen metabolism. The expression levels of substance P in the peripheral nerves and neutral endopeptidase, which degrades substance P, in the sebaceous glands of the facial skin are higher in patients with acne than in healthy individuals (72). However, the interaction between peripheral nerves in the EIME of the skin in acne remains poorly investigated.

In acne, factors other than the five major components of EIME, such as pre-adipocytes and triggering receptors expressed on myeloid cells 2 (TREM2)^+^ macrophages, have also been suggested (73, 74). Their interplay in the EIME is also expected to be critical for the pathogenesis and treatment of acne.

Thus, more than one inflammatory loops involving the pilosebaceous unit including sebocytes, *C. acnes*, and immune cells, emerge in the EIME of the skin in patients with acne vulgaris (**Figure 2E**), and may be primarily triggered by hormonal changes that influence sebum composition during puberty.

## 7 Discussion and concluding remarks

The epithelium senses external factors on the body surface in the earliest stages, determines the type of immune response, and constructs an optimal EIME that is best suited for defense. Epithelial stem cells memorize tissue invasion of the skin and respond rapidly to a second attack (75). This mechanism also induces allergic inflammation in the respiratory tract (76).

If chronic inflammation is a pathological mimic of host defense, the epithelium could also determine the type of inflammation in chronic inflammatory diseases by constructing each EIME, such as type 2 EIME in atopic dermatitis and type 17 EIME in psoriasis. This perspective raises several questions: What determines whether immune responses terminating in healthy skin are also terminated in chronic inflammatory diseases? What mechanisms of the epithelium determine the type of immune response? Do EIME in other epithelial organs, such as the gut and lungs, share common or unique mechanisms that govern biological defense and chronic inflammation?

The EIME concept will facilitate the development of new therapeutic targets for chronic inflammatory diseases because it simplifies the model of each disease. In addition, drawing the EIMEs for multiple diseases will clarify the contradictions involved in each existing model. Targeting disease-specific interrelationships between immune cells and non-immune factors will lead to the development of new therapies in the future.

## Author contributions

TD: Writing – original draft, Writing – review & editing. MI: Writing – review & editing.
